# 1,1′-(Ethane-1,2-di­yl)dipyridinium dichromate(VI)

**DOI:** 10.1107/S1600536812005430

**Published:** 2012-02-17

**Authors:** Mostafa Gholizadeh, Mehrdad Pourayoubi, Mehdi Kia, Arnold L. Rheingold, James A. Golen

**Affiliations:** a Department of Chemistry, Ferdowsi University of Mashhad, Mashhad 91779, Iran; b Department of Chemistry, Sabzevar Tarbiat Moallem University, Sabzevar, Iran; c Department of Chemistry, University of California, San Diego, 9500 Gilman Drive, La Jolla, CA 92093, USA

## Abstract

In the cation of the title salt, (C_12_H_14_N_2_)[Cr_2_O_7_], the two pyridinium moieties are in an *anti* orientation with respect to one another. The dihedral angle between the pyridine rings is 6.3 (2)°. The N—C—C—N torsion angle is 177.5 (2)°. In the dianion, the Cr^VI^ ions are in a slightly distorted tetra­hedral coordination environment and the bond angles at the independent Cr^VI^ ions are in the ranges 105.93 (10)–110.60 (11) and 107.35 (11)–111.07 (12)°. The Cr—O—Cr angle is 127.96 (12)°. The crystal used was an inversion twin with refined components of 0.510 (19) and 0.490 (19).

## Related literature

For the crystal structures of the salts with formula [C_5_H_5_NCH_2_CH_2_NC_5_H_5_]^2+^·2*X* ^−^ [*X* ^−^ = IO_3_^−^, IO_4_^−^], the preparation of 1,1′-(ethane-1,2-di­yl)dipyridinium dibromide and the orientation of pyridine moieties, see: Gholizadeh, Maleki *et al.* (2011 ▶); Gholizadeh, Hojati *et al.* (2011 ▶). For dichromate salts, see: Lennartson & Håkansson (2009 ▶); Averbuch-Pouchot *et al.* (1984 ▶).
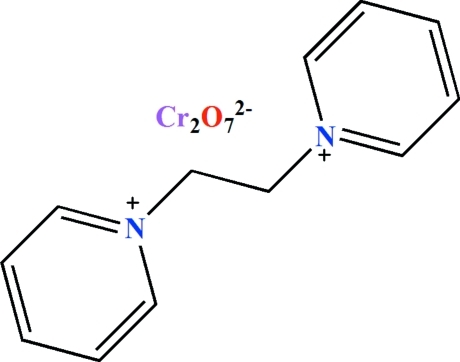


## Experimental

### 

#### Crystal data

(C_12_H_14_N_2_)[Cr_2_O_7_]
*M* *_r_* = 402.25
Monoclinic, 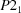

*a* = 8.2882 (4) Å
*b* = 9.0722 (4) Å
*c* = 10.0179 (5) Å
β = 91.882 (1)°
*V* = 752.86 (6) Å^3^
*Z* = 2
Mo *K*α radiation
μ = 1.48 mm^−1^
*T* = 100 K
0.22 × 0.15 × 0.15 mm

#### Data collection

Bruker APEXII CCD diffractometer
Absorption correction: multi-scan (*SADABS*; Bruker, 2005 ▶) *T*_min_ = 0.737, *T*_max_ = 0.808
5446 measured reflections
2628 independent reflections
2546 reflections with *I* > 2σ(*I*)
*R*_int_ = 0.025

#### Refinement

*R*[*F* ^2^ > 2σ(*F* ^2^)] = 0.027
*wR*(*F* ^2^) = 0.067
*S* = 1.04
2628 reflections
209 parameters
1 restraint
H-atom parameters constrained
Δρ_max_ = 0.47 e Å^−3^
Δρ_min_ = −0.21 e Å^−3^
Absolute structure: Flack (1983 ▶), 1163 Friedel pairs
Flack parameter: 0.510 (19)

### 

Data collection: *APEX2* (Bruker, 2005 ▶); cell refinement: *SAINT* (Bruker, 2005 ▶); data reduction: *SAINT*; program(s) used to solve structure: *SHELXS97* (Sheldrick, 2008 ▶); program(s) used to refine structure: *SHELXL97* (Sheldrick, 2008 ▶); molecular graphics: *SHELXTL* (Sheldrick, 2008 ▶); software used to prepare material for publication: *SHELXTL* and *enCIFer* (Allen *et al.*, 2004 ▶).

## Supplementary Material

Crystal structure: contains datablock(s) I, global. DOI: 10.1107/S1600536812005430/lh5410sup1.cif

Structure factors: contains datablock(s) I. DOI: 10.1107/S1600536812005430/lh5410Isup2.hkl

Additional supplementary materials:  crystallographic information; 3D view; checkCIF report

## Figures and Tables

**Fig. 1.**
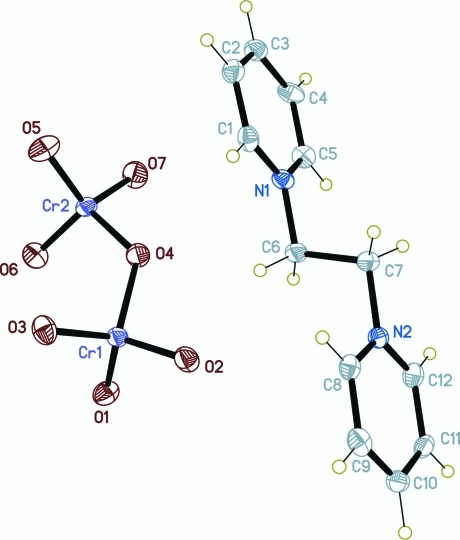
The molecular structure of the title compound. Ellipsoids are given at the 50% probability level.

## supplementary crystallographic information

### Comment

In recently published papers, the structure determinations of [C_5_H_5_NCH_2_CH_2_NC_5_H_5_]^2+^.2*X*^-^ [*X*^-^ = IO_3_^-^ (Gholizadeh, Maleki *et al.*, 2011) and IO_4_^-^ (Gholizadeh, Hojati *et al.*, 2011)] have been investigated. Structure determination of the title salt, [C_5_H_5_NCH_2_CH_2_NC_5_H_5_]^2+^.Cr_2_O_7_^2-^ (Fig. 1), was performed as a part of a project on the synthesis of a new hybrid compound containing an organic cation and an inorganic oxidant anion.

In the dication, two pyridinium moieties are *anti*-oriented with respect to one another similar to those observed in the 1,1'-(ethane-1,2-diyl)dipyridinium salts with iodate and periodate counter ions (Gholizadeh, Maleki *et al.*, 2011; Gholizadeh, Hojati *et al.*, 2011). In the dianion, each Cr^VI^ ion is in a slightly distorted tetrahedral coordination environment. The two pyridinium fragments in the cation and the two CrO_3_ units in the anion are not symmetrically equivalent.

The Cr—O bonds (with lengths of 1.777 (2) & 1.790 (2) Å) in the Cr—O—Cr fragment are longer than the other Cr—O bonds (in the range of 1.611 (2) to 1.624 (2) Å). The bond lengths and angles in the dichromate anion are within the expected values in the reported dichromate salts (Lennartson & Håkansson, 2009; Averbuch-Pouchot *et al.*, 1984).

### Experimental

1,1'-(ethane-1,2-diyl)dipyridinium dibromide was prepared according to the procedure reported by Gholizadeh, Maleki *et al.*, 2011 and Gholizadeh, Hojati *et al.*, 2011.

Preparation of title salt: To a solution of 1,1'-(ethane-1,2-diyl)dipyridinium dibromide (10 mmol) in H_2_O (25 ml), a solution of K_2_Cr_2_O_7_ (10 mmol) in H_2_O was added and stirred. After 1 h, the precipitate was filtered and washed with H_2_O. Orange crystals, suitable for X-ray crystallography, were obtained from a solution of the title salt in H_2_O at room temperature.

### Refinement

All H atoms were placed in their calculated positions and then refined using the riding model with atom—H lengths of 0.950 Å (CH) or 0.990 Å (CH_2_). Isotropic displacement parameters for these atoms were set to 1.20 times *U*_eq_ of the parent atoms.

### Crystal data

**Table:**

(C_12_H_14_N_2_)[Cr_2_O_7_]	*F*(000) = 408
*M**_r_* = 402.25	*D*_x_ = 1.774 Mg m^−^^3^
Monoclinic, *P*2_1_	Mo *K*α radiation, λ = 0.71073 Å
Hall symbol: P 2yb	Cell parameters from 3255 reflections
*a* = 8.2882 (4) Å	θ = 2.5–26.5°
*b* = 9.0722 (4) Å	µ = 1.48 mm^−^^1^
*c* = 10.0179 (5) Å	*T* = 100 K
β = 91.882 (1)°	Block, orange
*V* = 752.86 (6) Å^3^	0.22 × 0.15 × 0.15 mm
*Z* = 2	

### Data collection

**Table:**

Bruker APEXII CCD diffractometer	2628 independent reflections
Radiation source: fine-focus sealed tube	2546 reflections with *I* > 2σ(*I*)
Graphite monochromator	*R*_int_ = 0.025
φ and ω scans	θ_max_ = 25.3°, θ_min_ = 2.0°
Absorption correction: multi-scan (*SADABS*; Bruker, 2005)	*h* = −9→9
*T*_min_ = 0.737, *T*_max_ = 0.808	*k* = −10→10
5446 measured reflections	*l* = −12→12

### Refinement

**Table:**

Refinement on *F*^2^	Secondary atom site location: difference Fourier map
Least-squares matrix: full	Hydrogen site location: inferred from neighbouring sites
*R*[*F*^2^ > 2σ(*F*^2^)] = 0.027	H-atom parameters constrained
*wR*(*F*^2^) = 0.067	*w* = 1/[σ^2^(*F*_o_^2^) + (0.0332*P*)^2^ + 0.2849*P*] where *P* = (*F*_o_^2^ + 2*F*_c_^2^)/3
*S* = 1.04	(Δ/σ)_max_ = 0.001
2628 reflections	Δρ_max_ = 0.47 e Å^−^^3^
209 parameters	Δρ_min_ = −0.21 e Å^−^^3^
1 restraint	Absolute structure: Flack (1983), 1163 Friedel pairs
Primary atom site location: structure-invariant direct methods	Flack parameter: 0.510 (19)

### Special details

**Table:**

Geometry. All e.s.d.'s (except the e.s.d. in the dihedral angle between two l.s. planes) are estimated using the full covariance matrix. The cell e.s.d.'s are taken into account individually in the estimation of e.s.d.'s in distances, angles and torsion angles; correlations between e.s.d.'s in cell parameters are only used when they are defined by crystal symmetry. An approximate (isotropic) treatment of cell e.s.d.'s is used for estimating e.s.d.'s involving l.s. planes.
Refinement. Refinement of *F*^2^ against ALL reflections. The weighted *R*-factor *wR* and goodness of fit *S* are based on *F*^2^, conventional *R*-factors *R* are based on *F*, with *F* set to zero for negative *F*^2^. The threshold expression of *F*^2^ > σ(*F*^2^) is used only for calculating *R*-factors(gt) *etc*. and is not relevant to the choice of reflections for refinement. *R*-factors based on *F*^2^ are statistically about twice as large as those based on *F*, and *R*- factors based on ALL data will be even larger.

### Fractional atomic coordinates and isotropic or equivalent isotropic displacement parameters (Å^2^)

**Table:**

	*x*	*y*	*z*	*U*_iso_*/*U*_eq_	
Cr1	0.65538 (5)	0.04259 (4)	0.83530 (4)	0.01584 (13)	
Cr2	0.35152 (5)	−0.03987 (5)	0.64354 (4)	0.01691 (13)	
O1	0.7936 (2)	0.0097 (2)	0.7300 (2)	0.0250 (5)	
O2	0.7001 (2)	0.1896 (2)	0.9204 (2)	0.0230 (5)	
O3	0.6343 (3)	−0.0968 (2)	0.9351 (2)	0.0241 (5)	
O4	0.4684 (3)	0.0804 (2)	0.7502 (2)	0.0255 (5)	
O5	0.2177 (3)	−0.1216 (3)	0.7304 (2)	0.0312 (6)	
O6	0.4687 (3)	−0.1613 (2)	0.5775 (2)	0.0251 (5)	
O7	0.2677 (3)	0.0602 (3)	0.5277 (2)	0.0294 (5)	
N1	0.2884 (3)	0.3914 (3)	0.6933 (2)	0.0177 (6)	
N2	0.6566 (3)	0.5863 (3)	0.8330 (2)	0.0165 (6)	
C1	0.1985 (4)	0.3157 (4)	0.7779 (3)	0.0207 (7)	
H1	0.2448	0.2834	0.8608	0.025*	
C2	0.0391 (4)	0.2840 (3)	0.7460 (3)	0.0213 (7)	
H2	−0.0241	0.2291	0.8058	0.026*	
C3	−0.0274 (4)	0.3334 (4)	0.6255 (3)	0.0218 (7)	
H3	−0.1367	0.3118	0.6015	0.026*	
C4	0.0658 (4)	0.4138 (4)	0.5407 (3)	0.0224 (7)	
H4	0.0204	0.4503	0.4589	0.027*	
C5	0.2256 (4)	0.4412 (4)	0.5753 (3)	0.0208 (7)	
H5	0.2913	0.4949	0.5164	0.025*	
C6	0.4621 (4)	0.4188 (3)	0.7282 (3)	0.0193 (7)	
H6A	0.5255	0.4160	0.6461	0.023*	
H6B	0.5033	0.3406	0.7891	0.023*	
C7	0.4825 (3)	0.5674 (4)	0.7949 (3)	0.0192 (7)	
H7A	0.4468	0.6467	0.7328	0.023*	
H7B	0.4164	0.5724	0.8753	0.023*	
C8	0.7158 (3)	0.5188 (3)	0.9456 (3)	0.0179 (6)	
H8	0.6471	0.4605	0.9983	0.022*	
C9	0.8761 (4)	0.5360 (4)	0.9823 (3)	0.0217 (6)	
H9	0.9189	0.4901	1.0611	0.026*	
C10	0.9750 (4)	0.6206 (4)	0.9038 (3)	0.0219 (7)	
H10	1.0858	0.6328	0.9285	0.026*	
C11	0.9116 (4)	0.6876 (4)	0.7886 (3)	0.0197 (7)	
H11	0.9783	0.7456	0.7339	0.024*	
C12	0.7493 (4)	0.6681 (4)	0.7551 (3)	0.0197 (7)	
H12	0.7039	0.7129	0.6768	0.024*	

### Atomic displacement parameters (Å^2^)

**Table:**

	*U*^11^	*U*^22^	*U*^33^	*U*^12^	*U*^13^	*U*^23^
Cr1	0.0149 (2)	0.0163 (3)	0.0162 (2)	0.0001 (2)	−0.00137 (17)	−0.0007 (2)
Cr2	0.0149 (2)	0.0192 (3)	0.0165 (2)	−0.0025 (2)	−0.00182 (17)	0.0006 (2)
O1	0.0229 (11)	0.0267 (13)	0.0256 (11)	−0.0042 (9)	0.0042 (9)	−0.0029 (10)
O2	0.0232 (12)	0.0214 (12)	0.0239 (13)	0.0036 (9)	−0.0064 (9)	−0.0039 (10)
O3	0.0295 (12)	0.0219 (12)	0.0210 (11)	0.0040 (10)	0.0034 (9)	0.0030 (10)
O4	0.0215 (11)	0.0208 (12)	0.0333 (13)	−0.0003 (9)	−0.0105 (9)	−0.0024 (10)
O5	0.0265 (13)	0.0378 (15)	0.0296 (13)	−0.0101 (11)	0.0055 (10)	−0.0017 (11)
O6	0.0278 (12)	0.0271 (13)	0.0206 (11)	0.0027 (10)	0.0020 (9)	−0.0025 (10)
O7	0.0308 (12)	0.0277 (13)	0.0289 (11)	0.0000 (10)	−0.0100 (9)	0.0036 (11)
N1	0.0158 (13)	0.0200 (14)	0.0172 (12)	0.0023 (11)	−0.0016 (10)	−0.0032 (11)
N2	0.0164 (13)	0.0161 (14)	0.0171 (12)	0.0004 (10)	0.0018 (10)	−0.0037 (11)
C1	0.0259 (17)	0.0168 (16)	0.0189 (15)	0.0023 (14)	−0.0047 (12)	−0.0011 (14)
C2	0.0220 (16)	0.0193 (16)	0.0227 (16)	−0.0006 (13)	0.0002 (12)	0.0022 (14)
C3	0.0180 (16)	0.0227 (17)	0.0242 (16)	0.0007 (13)	−0.0032 (12)	−0.0044 (14)
C4	0.0187 (16)	0.0319 (19)	0.0167 (14)	0.0066 (14)	−0.0010 (12)	0.0004 (14)
C5	0.0230 (16)	0.0236 (18)	0.0160 (14)	0.0010 (14)	0.0025 (11)	0.0007 (14)
C6	0.0151 (14)	0.0199 (16)	0.0226 (15)	0.0003 (12)	−0.0034 (12)	−0.0034 (13)
C7	0.0148 (15)	0.0204 (16)	0.0224 (15)	0.0001 (13)	0.0002 (11)	0.0009 (14)
C8	0.0228 (15)	0.0164 (16)	0.0147 (14)	−0.0005 (13)	0.0029 (11)	−0.0033 (12)
C9	0.0294 (16)	0.0213 (16)	0.0142 (13)	0.0052 (15)	−0.0021 (11)	−0.0054 (14)
C10	0.0174 (15)	0.0215 (17)	0.0267 (17)	0.0018 (14)	−0.0025 (12)	−0.0098 (15)
C11	0.0198 (15)	0.0161 (15)	0.0234 (17)	−0.0040 (12)	0.0037 (12)	−0.0034 (13)
C12	0.0241 (17)	0.0182 (16)	0.0169 (15)	−0.0010 (14)	0.0009 (12)	−0.0027 (13)

### Geometric parameters (Å, º)

**Table:**

Cr1—O1	1.611 (2)	C3—H3	0.9500
Cr1—O2	1.620 (2)	C4—C5	1.380 (4)
Cr1—O3	1.624 (2)	C4—H4	0.9500
Cr1—O4	1.777 (2)	C5—H5	0.9500
Cr2—O5	1.612 (2)	C6—C7	1.511 (4)
Cr2—O7	1.613 (2)	C6—H6A	0.9900
Cr2—O6	1.624 (2)	C6—H6B	0.9900
Cr2—O4	1.790 (2)	C7—H7A	0.9900
N1—C1	1.337 (4)	C7—H7B	0.9900
N1—C5	1.353 (4)	C8—C9	1.376 (4)
N1—C6	1.491 (4)	C8—H8	0.9500
N2—C12	1.338 (4)	C9—C10	1.386 (4)
N2—C8	1.361 (4)	C9—H9	0.9500
N2—C7	1.491 (3)	C10—C11	1.392 (4)
C1—C2	1.379 (4)	C10—H10	0.9500
C1—H1	0.9500	C11—C12	1.387 (4)
C2—C3	1.385 (4)	C11—H11	0.9500
C2—H2	0.9500	C12—H12	0.9500
C3—C4	1.376 (5)		
			
O1—Cr1—O2	110.01 (11)	N1—C5—C4	119.8 (3)
O1—Cr1—O3	110.60 (11)	N1—C5—H5	120.1
O2—Cr1—O3	110.15 (11)	C4—C5—H5	120.1
O1—Cr1—O4	110.43 (11)	N1—C6—C7	110.2 (2)
O2—Cr1—O4	105.93 (10)	N1—C6—H6A	109.6
O3—Cr1—O4	109.62 (11)	C7—C6—H6A	109.6
O5—Cr2—O7	111.07 (12)	N1—C6—H6B	109.6
O5—Cr2—O6	109.80 (13)	C7—C6—H6B	109.6
O7—Cr2—O6	109.78 (12)	H6A—C6—H6B	108.1
O5—Cr2—O4	109.08 (11)	N2—C7—C6	108.0 (2)
O7—Cr2—O4	107.35 (11)	N2—C7—H7A	110.1
O6—Cr2—O4	109.72 (10)	C6—C7—H7A	110.1
Cr1—O4—Cr2	127.96 (12)	N2—C7—H7B	110.1
C1—N1—C5	121.2 (3)	C6—C7—H7B	110.1
C1—N1—C6	119.4 (2)	H7A—C7—H7B	108.4
C5—N1—C6	119.3 (3)	N2—C8—C9	119.3 (3)
C12—N2—C8	122.4 (2)	N2—C8—H8	120.4
C12—N2—C7	119.0 (2)	C9—C8—H8	120.4
C8—N2—C7	118.7 (2)	C8—C9—C10	119.6 (3)
N1—C1—C2	120.7 (3)	C8—C9—H9	120.2
N1—C1—H1	119.7	C10—C9—H9	120.2
C2—C1—H1	119.7	C9—C10—C11	119.9 (3)
C1—C2—C3	119.0 (3)	C9—C10—H10	120.1
C1—C2—H2	120.5	C11—C10—H10	120.1
C3—C2—H2	120.5	C12—C11—C10	118.8 (3)
C4—C3—C2	119.6 (3)	C12—C11—H11	120.6
C4—C3—H3	120.2	C10—C11—H11	120.6
C2—C3—H3	120.2	N2—C12—C11	120.0 (3)
C3—C4—C5	119.6 (3)	N2—C12—H12	120.0
C3—C4—H4	120.2	C11—C12—H12	120.0
C5—C4—H4	120.2		
			
O1—Cr1—O4—Cr2	62.94 (19)	C1—N1—C6—C7	−94.5 (3)
O2—Cr1—O4—Cr2	−177.98 (15)	C5—N1—C6—C7	86.8 (3)
O3—Cr1—O4—Cr2	−59.16 (19)	C12—N2—C7—C6	100.0 (3)
O5—Cr2—O4—Cr1	94.27 (18)	C8—N2—C7—C6	−79.6 (3)
O7—Cr2—O4—Cr1	−145.29 (16)	N1—C6—C7—N2	177.5 (2)
O6—Cr2—O4—Cr1	−26.04 (19)	C12—N2—C8—C9	0.7 (4)
C5—N1—C1—C2	1.1 (5)	C7—N2—C8—C9	−179.7 (3)
C6—N1—C1—C2	−177.6 (3)	N2—C8—C9—C10	−0.5 (5)
N1—C1—C2—C3	−0.8 (5)	C8—C9—C10—C11	0.0 (5)
C1—C2—C3—C4	−0.5 (5)	C9—C10—C11—C12	0.2 (5)
C2—C3—C4—C5	1.6 (5)	C8—N2—C12—C11	−0.5 (5)
C1—N1—C5—C4	0.0 (5)	C7—N2—C12—C11	179.9 (3)
C6—N1—C5—C4	178.7 (3)	C10—C11—C12—N2	0.1 (5)
C3—C4—C5—N1	−1.3 (5)		
